# Nanodiamonds Co-Localize with *Mycobacterium tuberculosis* in Foamy Macrophages of Infected Mouse Lungs

**DOI:** 10.3390/pharmaceutics18060671

**Published:** 2026-05-29

**Authors:** Maria V. Erokhina, Alexander G. Masyutin, Georgii V. Lisichkin, Pavel G. Mingalev, Gennadii A. Badun, Larisa N. Lepekha, Irina V. Bocharova, Ekaterina K. Tarasova, Atadzhan E. Ergeshov

**Affiliations:** 1 Department of Biology, Lomonosov Moscow State University, 1-12 Leninskie Gory, Moscow 119991, Russia; masha.erokhina@gmail.com; 2 Central Tuberculosis Research Institute, 2 Yauzskaya Alleya, Moscow 107564, Russia; lep3@yandex.ru (L.N.L.); 3595598@mail.ru (I.V.B.); tarasova.ek.nano@yandex.ru (E.K.T.); cniit@ctri.ru (A.E.E.); 3 Department of Chemistry, Lomonosov Moscow State University, 1-12 Leninskie Gory, Moscow 119234, Russia; lisich@petrol.chem.msu.ru (G.V.L.); uuk2@mail.ru (P.G.M.); badunga@my.msu.ru (G.A.B.)

**Keywords:** nanodiamonds, tuberculosis, *M. tuberculosis*, lungs, macrophages, co-localization, electron microscopy

## Abstract

**Background:** Pulmonary tuberculosis (TB) is an infectious disease caused by *Mycobacterium tuberculosis* (*M. tuberculosis*). Drug-resistant TB remains a major public health challenge and calls for new approaches to drug development. Targeted delivery of antibacterial agents using nanoscale carriers represents one such approach. A decisive factor for efficient targeting is the judicious selection of the carrier platform. **Methods:** In the present study, diamond nanoparticles were evaluated as a prospective vehicle for conveying anti-TB drugs to lung cells. Conventional and analytical transmission electron microscopy were used to analyze the localization of the nanodiamonds (NDs) in the lungs of *M. tuberculosis*-infected mice 30 days after nanoparticle administration and 44 days post-infection. **Results:** The study shows that the NDs co-localize with *M. tuberculosis* in foamy macrophages of the lung, residing in the same cellular compartments—phagosomes/phagolysosomes and lipid droplets. These in vivo results demonstrate a high degree of macrophage-specific accumulation of NDs relative to *M. tuberculosis*. **Conclusions:** Consequently, NDs can be considered a promising carrier for targeted delivery of anti-TB therapeutics to the lungs during TB-induced inflammation.

## 1. Introduction

Pulmonary TB is an infectious disease that has afflicted humankind for centuries and continues to pose a threat to public health today [1,2]. TB is prevalent in every country and affects people of all age groups; the global burden in 2024 is estimated at 10.7 million cases. Drug-resistant TB, in particular, represents a major challenge for public health. In 2024, only two out of every five patients with drug-resistant tuberculosis were able to obtain treatment [3]. In this context, interest in targeted drug delivery—in which active pharmaceutical compounds are immobilized on a carrier and conveyed directly to the diseased organ and cellular targets—has risen markedly in recent years. According to the records of PubMed Central, 1362 papers on this topic were published in 2015, whereas by 2025 the number had increased to 10,752, representing almost an eight-fold expansion [4].

Targeted delivery of therapeutic agents via nanocarriers can increase the drug concentration in the target organ, extend and enhance the compound’s pharmacological efficacy, and simultaneously reduce adverse side effects [5]. This innovative approach is already being incorporated into the therapy of diseases of diverse etiologies, each year, additional nanotechnology-based medicines gain approval from the Food and Drug Administration. Since 1995, more than 90 clinically used medicinal products based on liposomes, nanocrystals, silver nanoparticles, and other nanoparticles/nanomaterials have been approved [6]. New antibacterial strategies based on nanoparticle-containing formulations and nanostructured surfaces are also being actively developed [7]. These approaches are particularly relevant for combating antibiotic-resistant bacterial strains, which represent an increasing challenge and a substantial burden for healthcare systems [8].

It is well established that direct interactions between antibacterial nanoparticles and the bacterial cell wall or membrane can increase permeability, disrupt membrane function, and promote the generation of intracellular reactive oxygen species. These reactive oxygen species, in turn, may destabilize ribosomal function and damage proteins and DNA, ultimately leading to bacterial death [9]. Thus, nanoformulations may bypass conventional bacterial defense and resistance mechanisms and effectively target complex bacterial communities, including biofilms. The development of multidrug resistance and extensive drug resistance, including through biofilm formation, is also characteristic of *M. tuberculosis*. This highlights the relevance of developing new nanocarrier-based formulations of anti-tuberculosis drugs. The tunable physicochemical properties of nanocarriers enable targeted drug delivery and multimodal mechanisms of action, thereby potentially reducing the risk of resistance development at an early stage [10]. Within the development of new anti-TB drug formulations, the use of nanocarriers is likewise regarded as a promising avenue [11].

One of the key determinants of effective targeted delivery is the judicious choice of the carrier platform. The carrier must satisfy several criteria—biosafety and biocompatibility, size, shape, surface charge, hydrophobic or hydrophilic character, and amenability to surface functionalization. Nanodiamonds (NDs) meet these requirements: they are nanoscale (4–100 nm), quasi-spherical particles composed of a diamond core encased in a graphitic shell endowed with numerous free chemical bonds. Relative to other platforms, NDs exhibit low cytotoxicity [12,13] and possess a chemically labile surface that can be functionalized (e.g., by hydrogenation or carboxylation) to accommodate pharmaceutical and bioactive molecules [14]. A further advantageous property of NDs is their ability to accumulate intracellularly within pulmonary macrophages.

Previously, we demonstrated in vitro that human macrophages internalize NDs via two distinct mechanisms. The first involves the formation of filopodia, whereas the second entails the generation of plasma–membrane invaginations. Typically, these were small ND aggregates up to 100 nm in size [15]. In vitro, once engulfed by macrophages, NDs are localized within phagosomes/phagolysosomes. In mouse lungs, seven days after a single administration, NDs were detected in endothelial cells and interstitial macrophages. This finding indicates that endothelial cells sequester NDs from the bloodstream and subsequently transfer them into the alveolar interstitium (via transcytosis), where they are taken up by interstitial macrophages [15]. NDs labeled with radioactive iodine exhibit high accumulation in the lungs as early as 30 min after intravenous injection, and this retention persists for up to 28 days following a single dose [16]. Pulmonary accumulation of NDs in animals has likewise been reported by other investigators [13,16,17]. For instance, a 2009 study on ND accumulation, clearance, and toxicity in mouse lungs [18] employed NDs of 4 nm and 50 nm diameter. Both ND variants proved non-toxic upon intratracheal administration and remained detectable in the lungs 28 days post-exposure without eliciting pathological changes in surrounding cells or tissues. Collectively, these observations indicate that NDs constitute a promising platform for drugs intended to treat chronic pulmonary diseases, including pulmonary TB.

For a rigorous evaluation of the precision and efficiency of targeted nano-enabled anti-TB drug delivery to the lungs, it is necessary to demonstrate not only that the carrier platform is detected and accumulates in the target organ and cell types, but also that it is co-localized with the tuberculosis pathogen *M. tuberculosis*. One of the major challenges when working with biomedical nanoparticles in vivo is their reliable detection and unequivocal identification within the target cells and organ. Nanoparticles may undergo biotransformation, such as acquisition of a protein corona, formation of morphologically indistinguishable agglomerates, or partial biodegradation [19,20,21,22]. Therefore, when assessing their co-localization with *M. tuberculosis*, special care must be taken to identify NDs accurately in order to avoid false-positive results.

Analytical electron microscopy [23] is one of the principal techniques for identifying nanoparticles in biological material. Because NDs measure only 4–5 nm, their detection and unambiguous identification within electron-dense biological matrices are challenging. In our previous work, we identified NDs in mouse lungs by electron diffraction analysis and by limiting contrast enhancement during tissue processing—using osmium tetroxide and uranyl acetate during embedding but omitting post-section staining with lead citrate—which enabled us to distinguish NDs from other nanoscale electron-dense structures in cells [15]. The aim of the present study was to detect and identify NDs and to establish their co-localization with *Mycobacterium tuberculosis* (strain H37Rv) in the lungs of infected mice. NDs were administered 14 days after infection, and their detection together with co-localization analysis was performed 30 days after administration. Ultrastructural examination of cells and tissues from *M. tuberculosis*-infected mice, as well as ND detection and identification, were carried out using conventional transmission electron microscopy (TEM), analytical electron microscopy (electron diffraction mode), and high-resolution TEM (HRTEM).

## 2. Materials and Methods

### 2.1. Nanodiamonds

Detonation-synthesized nanodiamond particles used in this study were supplied by Tekhnolog Company (St. Petersburg, Russia). The stock concentration was 4 mg mL^−1^. According to the supplier, the nanodiamonds suspended in water have a nominal diameter of 4–6 nm. We employed hydrogenated NDs, which display hydrophobic surface properties, because many anti-tuberculosis drugs used clinically are likewise hydrophobic [24]. Surface hydrogenation of the nanodiamonds was carried out as previously described [25]. DND powder was subjected to hydrogen treatment by exposing a 500 mg sample to a hydrogen flow of 2.5 L h^−1^ for 5 h at 800 °C; hydrogen gas with a purity of 99.99% was used. For oxidative acid treatment, 75 mL of a concentrated HNO_3_/H_2_SO_4_ mixture, prepared at a volume ratio of 1:9, was placed in a 150 mL flat-bottomed glass flask equipped with a reflux condenser. DND powder, 1 g, was then added, and the resulting suspension was heated at 120 °C for 24 h under constant stirring.

The resulting nanomaterial forms an opaque, bluish-gray hydrosol.

### 2.2. Infection and Nanoparticle Administration in Mice

Female BALB/c mice aged 10–12 weeks and weighing 22–23 g were used in the study. Animals were group-housed in a standard vivarium under controlled environmental conditions, including a 12 h light/dark cycle, temperature of 20–24 °C, and relative humidity of 45–65%; food and water were provided ad libitum. Mice were acclimatized to vivarium conditions for at least two weeks before any experimental procedures.

A total of 21 animals were included in the experiment and allocated into three experimental groups, with seven animals per group: (1) *M. tuberculosis*-infected mice examined 44 days post-infection; (2) *M. tuberculosis*-infected mice examined 30 days after nanodiamond administration; and (3) uninfected mice examined 30 days after nanodiamond administration. Aerosol infection was performed using a Glas-Col inhalation exposure chamber (Glas-Col, Terre Haute, USA). Each mouse received an estimated inoculum of 100 CFU of M. tuberculosis strain H37Rv per lung; the strain was obtained from the Institut Pasteur. Aerosol infection is considered one of the most appropriate methods for modeling human *M. tuberculosis* infection in mice [26].

On day 14 post-infection, infected animals were divided into two groups: mice receiving a suspension of nanodiamonds (n = 7) and control mice receiving saline (n = 7). A single intravenous dose of the nanodiamond suspension (4 mg mL^−1^ in saline) was administered immediately after sonication (at 25 °C, power 90 W, Elma Ultrasonic, Singen, Germany). The suspension (200 µL, 0.8 mg per mouse) was injected slowly into the lateral tail vein. Mice were euthanized 30 days after ND administration (44 days after the start of the experiment). All procedures were carried out in the certified animal facility of the Central Research Institute of Tuberculosis (Moscow, Russia). *M. tuberculosis* cultures were grown on Middlebrook 7H9 medium (BD Difco, Franklin Lakes, NJ, USA) supplemented with 10% Middlebrook OADC, 0.02% glycerol, and 0.05% Tween 80 at 37 °C.

Animals were housed in accordance with Ministry of Health of the Russian Federation guideline No. 755 and the INH Office of Laboratory Animal Welfare (OLAW). All experimental procedures were approved by the institute’s Animal Care and Use Committee (IACUC protocols Nos. 2, 5, 6, 14; protocol No. 2 dated 2 February 2021).

### 2.3. Conventional Histology

Tissue samples were fixed in 10% buffered formalin (BioVitrum, Saint Petersburg, Russia). Histological specimens were dehydrated according to a standard protocol using a graded ethanol series from 70% to 96%; absolute ethanol was replaced with isopropanol. After treatment with isopropanol, the samples were embedded in paraffin (“Hystomix”, BioVitrum, Saint Petersburg, Russia). Sections 5 µm in thickness were prepared using a Leica microtome (Leica, Wetzlar, Germany). The sections were stained with hematoxylin (Karazzi method) and eosin (0.1% aqueous solution) and mounted in Canada balsam. Microscopic analysis was subsequently performed using a Leica DM 1000 LED light microscope equipped with N Plan objectives (4×, 20×, 40×, and 100×/1.25 oil).

Semithin sections (1 μm thick) were made using an LKB 11800 Pyramitome (LKB, Bromma, Sweden) and mounted onto glass slides. Sections were stained with an aqueous ethanol solution of azure B and basic fuchsin according to Morikawa et al. [27]. Microscopic analysis was subsequently performed using a Leica DM 1000 (Wetzlar, Germany) and Olympus BX43 light microscopes (Tokyo, Japan).

### 2.4. Electron Microscopy

To characterize the nanoparticles, an aqueous suspension of native NDs was drop-cast onto formvar-coated copper grids, air-dried, and examined by conventional transmission electron microscopy, high-resolution TEM (HRTEM), and electron diffraction analysis. For ultrastructural examination of *M. tuberculosis* cells and lung fragments from infected mice, samples were fixed in 2.5% glutaraldehyde (TedPella, Redding, CA, USA) in 0.1 M phosphate buffer for 2 h and post-fixed in 1% osmium tetroxide (OsO_4_) for 2 h at +4 °C. Dehydration was carried out in a graded ethanol series followed by acetone according to a standard protocol [28]. After dehydration, the specimens were embedded in epoxy resin. Sections were cut on a Leica UCT-4 ultra microtome (Leica, Wetzlar, Germany) and stained by the Reynolds method [29].

Ultrathin sections of cells and native nanoparticles were analyzed with a JEM-2100 transmission electron microscope operating at 200 kV (non-corrected, LaB_6_ cathode; JEOL, Tokyo, Japan) equipped with a Gatan FT1000 2 k camera (Gatan, Pleasanton, CA, USA). A camera length of 20 cm was used to obtain electron diffraction patterns from electron-dense inclusions within cells. Conventional TEM imaging was performed on a JEM-1400 microscope operating at 100 kV (JEOL, Tokyo, Japan).

### 2.5. Analysis of ND Accumulation in Lung Cells

Nanodiamond accumulation in lung cells was quantified using overview transmission electron microscopy (TEM) micrographs acquired from different regions of ultrathin sections, 60–80 nm in thickness. Micrographs with field sizes of 25 × 20 μm and 20 × 11 μm were obtained at magnifications of ×10,000, ×25,000, and ×50,000. For each micrograph, the following parameters were recorded: (i) the total number of cells and the proportion of foamy macrophages; (ii) the number of cells containing nanodiamonds; and (iii) the number of foamy macrophages containing *M. tuberculosis* and/or nanodiamonds. To improve counting accuracy, cells were identified according to nuclear localization and visible cell boundaries. Quantitative analysis was performed using two lung tissue fragments/blocks from each of seven infected animals.

### 2.6. Statistical Analysis

Statistical analysis was performed using GraphPad Prism 8 (GraphPad Software, Boston, MA, USA). Proportions were calculated as the number of cells with a given feature divided by the total number of cells in the corresponding category. Ninety-five percent confidence intervals for proportions were calculated using the Wilson score method. Associations between categorical variables were assessed using the two-sided Fisher’s exact test. These analyses were considered exploratory because pooled cell counts rather than animal-level means were used.

## 3. Results

**Figure 1:**
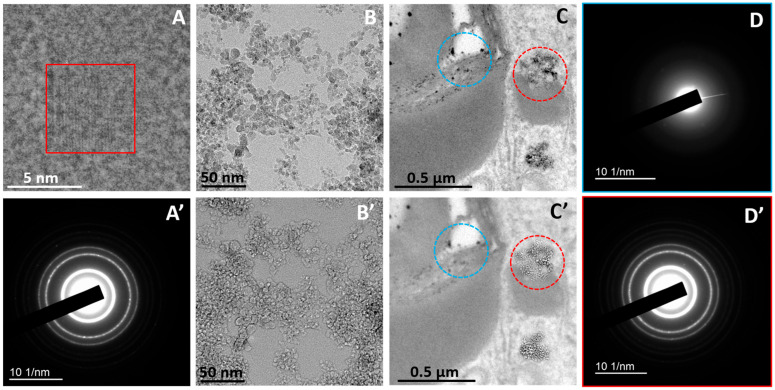
Visualization and intracellular identification of nanodiamonds: (**A**)—lattice fringes of pristine NDs (red frame), HRTEM; (**A’**)—reference electron diffraction pattern from pristine NDs, analytical TEM. (**B**,**B’**)—visualization of NDs through contrast changes produced by defocusing the electron beam; (**C**,**C’**)—use of defocus for rapid detection of NDs among electron-dense structures in mouse lung cells, conventional TEM. Nanodiamonds are outlined in red; electron-dense inclusions within a lipid droplet are outlined in blue. (**D**,**D’**)—electron diffraction patterns recorded from different electron-dense objects in a foamy macrophage of *M. tuberculosis*-infected mice.

The NDs used in this study are crystalline, as demonstrated by HRTEM, which reveals characteristic lattice fringes (Figure 1A) and by electron diffraction, which produces the distinctive ring pattern of NDs (Figure 1A’). Because of this crystallinity, applying defocus causes the nanoparticles to change from dark (Figure 1B) to bright contrast (Figure 1B’). This method allows a rapid, qualitative check of the crystallinity of electron-dense objects in cells and tissues before their chemical identity is confirmed by electron diffraction. Under the same defocus conditions, amorphous electron-dense structures appear unchanged, in contrast to NDs (Figure 1C,C’). Although many electron-dense inclusions in the lipid droplets of foamy macrophages from *M. tuberculosis*-infected mice look similar, only a subset are unequivocally NDs, as verified by their diffraction patterns; the remaining inclusions are amorphous, a conclusion likewise confirmed by electron diffraction (Figure 1D,D’).

**Figure 2:**
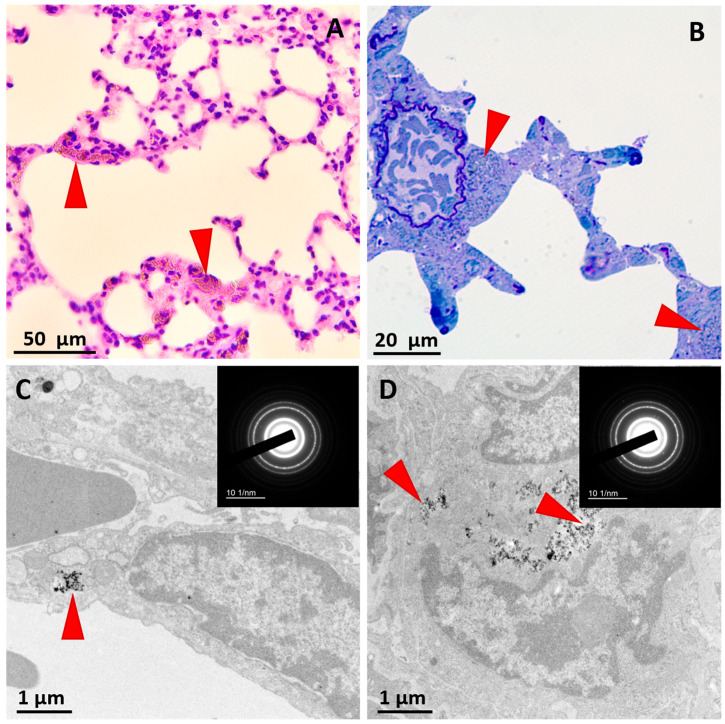
Detection and identification of ND aggregates in the lungs of uninfected mice 30 days after administration. (**A**)—General view of mouse lung alveoli: dark ND inclusions are detected in the alveolar walls; hematoxylin–eosin staining, light microscopy. (**B**)—Semithin section from the perivascular region of mouse lung: intracellular ND inclusions are detected; azure B and basic fuchsin staining, light microscopy; (**C**,**D**)—ND aggregates in endothelial cells (**C**) and a interstitial macrophages (**D**), conventional TEM. Insets show electron micrographs obtained by analytical TEM. Red arrows indicate ND aggregates in alveolar cells.

Analysis of ND localization in lung tissue sections stained with hematoxylin and eosin demonstrated the presence of nanoparticles in the alveolar walls in the form of dark inclusions (Figure 2A). Semithin sections showed ND accumulation in perivascular regions (Figure 2B). Additional examination using conventional and analytical TEM allowed precise identification of ND aggregates within phagosomes/phagolysosomes of endothelial cells (Figure 2C) and interstitial macrophages (Figure 2D).

**Figure 3:**
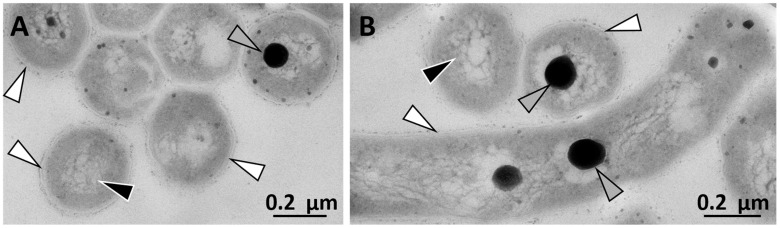
Ultrastructure of *M. tuberculosis*: (**A**)—transverse section, (**B**)—longitudinal section. White arrows indicate the multi-layered cell wall, black arrows the nucleoid, and gray arrows electron-dense inclusions within the cytoplasm. Conventional TEM.

For the subsequent localization of bacteria in mouse lung cells, we first obtained electron micrographs of *M. tuberculosis* ultrathin sections in vitro. The images clearly display the bacterium’s characteristic multi-layered cell wall, its nucleoid region, and electron-dense inclusions within the cytoplasm (Figure 3).

**Figure 4:**
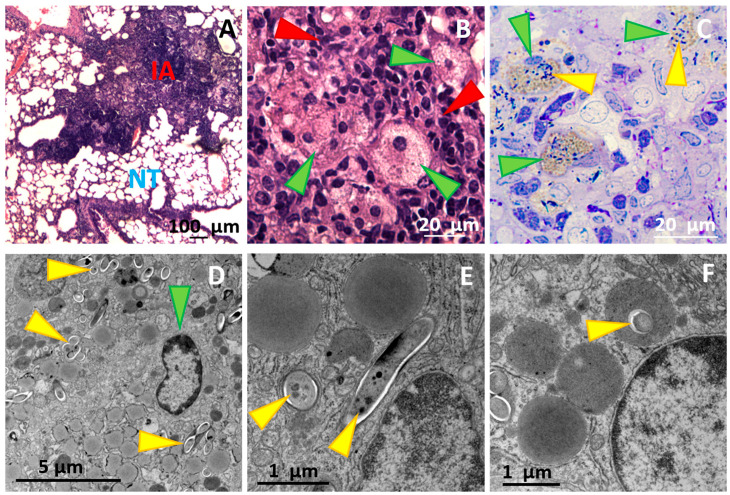
*M. tuberculosis* in lung macrophages during experimental tuberculous inflammation in mice. (**A**–**C**)—Inflammatory focus in mouse lungs after infection with *M. tuberculosis*; hematoxylin–eosin stain, light microscopy: (**A**)—inflammatory area (IA) surrounded by intact (normal) tissue (NT), 44 days post-infection; (**B**)—cluster of foamy macrophages (green arrow) and lymphocytes (red arrow) within the inflammatory zone. (**C**)—Semithin section showing a cluster of foamy macrophages containing *M. tuberculosis* (yellow arrows); azure B and basic fuchsin staining, light microscopy. (**D**–**F**)—Foamy macrophages in the lungs of *M. tuberculosis*-infected mice, conventional TEM: (**D**)—Overview of a foamy macrophage (green arrow); (**E**)—Longitudinal section of a single *M. tuberculosis* bacillus inside an infected macrophage; (**F**)—*M. tuberculosis* situated inside a lipid droplet of a foamy macrophage.

Histological examination of mouse lungs showed that, 44 days after infection, the respiratory portion contained inflammatory foci appearing as large infiltrates, whereas some alveoli remained aerated (Figure 4A). Within the inflammatory infiltrates, aggregates of lymphocytes and foamy macrophages are evident; the latter are large cells with round nuclei and a pale, vacuolated cytoplasm (Figure 4B). These cells measure approximately 20–30 μm in diameter and contain numerous phagosomes and lipid droplets, features that account for their characteristic “foamy” appearance (Figure 4C). Clusters of *M. tuberculosis* were observed specifically within the phagosomes of these cells (Figure 4C,D). Individual bacilli present as rod-shaped structures ~ 0.3 µm in diameter and up to 2.5 µm in length, with a clearly visible multi-layered cell wall; ribosomes and metabolic inclusions are discernible inside each bacterium (Figure 4E). *M. tuberculosis* was found within phagosomes (Figure 4D,E). In addition, individual *M. tuberculosis* cells were identified inside lipid droplets (Figure 4F).

**Figure 5:**
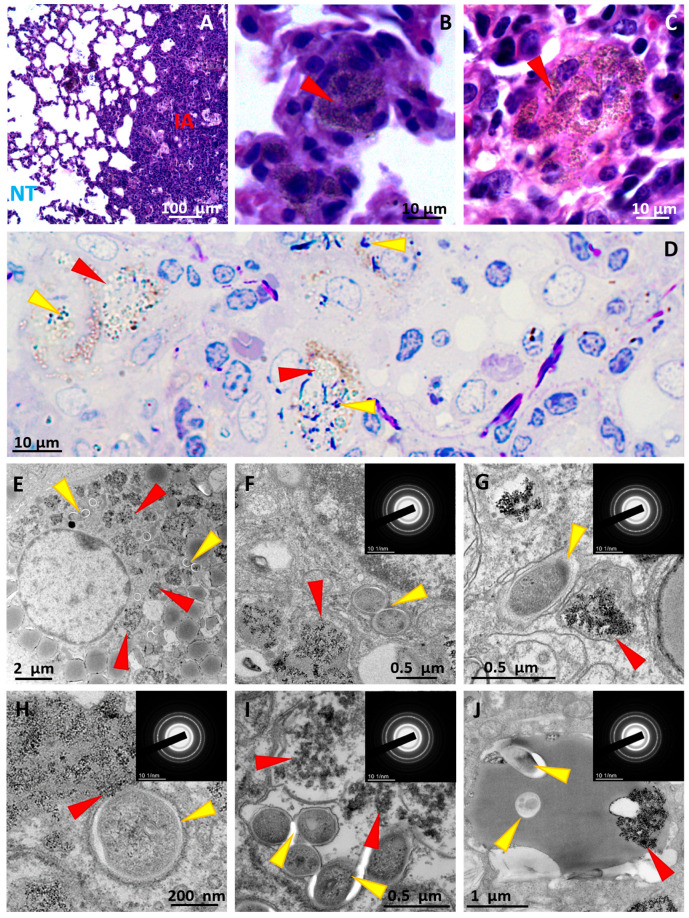
Identification ND aggregates and their co-localization with *M. tuberculosis* in foamy macrophages of mouse lungs during experimental tuberculous inflammation. (**A**–**C**)—Dark ND inclusions in the respiratory portion of the lung; hematoxylin–eosin stain, light microscopy: A—inflammatory area (IA) adjacent to intact tissue (NT); (**B**)—dark inclusions within interstitial macrophages located in the alveolar septum; (**C**)—dark inclusions within foamy macrophages (indicated by arrows); (**D**)—semithin section showing ND aggregates (red arrows) and *M. tuberculosis* bacilli (yellow arrows) within pulmonary foamy macrophages; azure B and basic fuchsin staining, light microscopy. (**E**–**J**)—Co-localization analysis of NDs with *M. tuberculosis*; conventional and analytical TEM: (**E**)—NDs and *M. tuberculosis* within a foamy macrophage; (**F**)—NDs and *M. tuberculosis* located in separate phagosomes; (**G**)—phagosomes containing NDs and *M. tuberculosis* lying in close apposition; (**H**)—fusion of the ND-containing phagosome with the *M. tuberculosis*-containing phagosome, (**I**)—NDs and *M. tuberculosis* localized together in a single phagosome, (**J**)—NDs and *M. tuberculosis* situated within the same lipid droplet. Red arrows mark ND clusters, yellow arrows mark *M. tuberculosis*. Insets show electron diffraction patterns recorded from the ND aggregates.

After the nanoparticle suspension was administered, we detected the particles in both intact lung tissue and areas of tuberculous inflammation (Figure 5A). Electron-dense aggregates are present in interstitial macrophages of the alveolar septa (Figure 5B), as well as in foamy macrophages (Figure 5C). Ultrastructural examination of inflamed regions showed that nanoparticles were confined to foamy macrophages and were absent from other cell types. Within these macrophages, numerous lipid inclusions coexist with clusters of nanoparticles and with *M. tuberculosis* (Figure 5D,E).

The NDs resided inside phagosomes, where they formed loose clusters. In situ, the particles displayed the same morphology as pristine NDs and were unambiguously identified by electron diffraction (Figure 5, insets).

We observed several spatial configurations of NDs and *M. tuberculosis* within foamy macrophages that were (i) located in separate, widely spaced phagosomes (Figure 5F); (ii) in neighboring phagosomes (Figure 5G); (iii) in merging phagosomes (Figure 5H); (iv) together within a single large phagosome (Figure 5I); (v) inside a lipid droplet (Figure 5J).

For quantitative assessment, cells were counted in the inflammatory region and in the adjacent local region of aerated alveoli. Inflammatory regions were first identified in semithin sections. TEM enabled the detection and identification of small nanoparticle aggregates up to 200 nm in size within cells, which were not visible by light microscopy. In TEM images of the lungs of infected mice, a total of 3586 cells were counted in inflammatory regions and 1240 cells in aerated alveolar regions.

**Figure 6:**
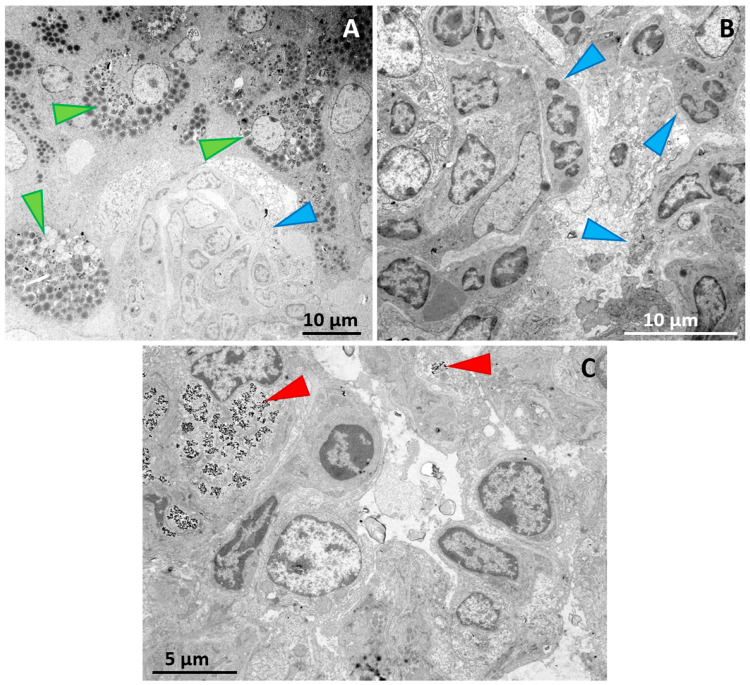
Inflammatory regions in the lungs of *M. tuberculosis* infected mice: (**A**)—cluster of foamy macrophages, containing *M. tuberculosis* and ND aggregates (green arrows) and cluster of inflammatory cells without NDs (blue arrow); (**B**)—inflammation area without foamy macrophages (blue arrows). No cells with internalized NDs or *M. tuberculosis* are detected. (**C**)—Cells containing ND aggregates (red arrows) in aerated alveolar regions. TEM images.

In inflammatory regions (examples shown in Figure 6A,B), foamy macrophages accounted for 11.8% of all counted cells, 95% CI: 10.8–12.9% (Table 1). Among foamy macrophages, 82.5% contained NDs, 95% CI: 78.6–85.9%, whereas 64.4% contained *M. tuberculosis*, 95% CI: 59.7–68.8%. Both NDs and *M. tuberculosis* were detected in 51.9% of foamy macrophages, 95% CI: 47.1–56.6%. Among *M. tuberculosis*-positive foamy macrophages, 80.6% also contained NDs, 95% CI: 75.5–84.8%. In aerated alveolar regions (example shown in Figure 6C), ND-positive cells accounted for 7.0% of all counted cells, 95% CI: 5.7–8.6%. No *M. tuberculosis*-positive cells were identified in these regions, 0/1240 cells, 95% CI: 0–0.31%.

As an exploratory cell-level analysis, a 2 × 2 contingency table was constructed to assess whether ND accumulation was associated with the visible presence of *M. tuberculosis* in foamy macrophages. Among *M. tuberculosis*-positive foamy macrophages, 220/273 cells, 80.6%, contained NDs, whereas among *M. tuberculosis*-negative foamy macrophages, 130/151 cells, 86.1%, contained NDs. This difference was not statistically significant (Fisher’s exact test, two-sided *p* = 0.182).

Thus, qualitative and quantitative analyses revealed that ND aggregates are localized in different cells in these two lung zones of infected mice. This important result demonstrates that, after entering the bloodstream, the nanoparticles reach the tuberculosis lesion and are subsequently detected in infected foamy macrophages, occupying the very intracellular compartments harboring *M. tuberculosis*.

In addition to the lungs, ND aggregates were detected in extrapulmonary organs of *M. tuberculosis*-infected animals, including splenic macrophages and both macrophages and hepatocytes in the liver (Figures S1 and S2).

## 4. Discussion

*M. tuberculosis* is an intracellular pathogen capable of persisting for extended periods within lung macrophages, remaining inside phagosomes or escaping into the cytoplasm as a consequence of phagosomal disruption [30,31,32]. Such intracellular localization of the pathogen markedly diminishes the efficacy of many antibiotics, thereby necessitating a specialized therapeutic approach to TB [33]. Accordingly, when selecting carriers for targeted drug delivery, it is crucial to evaluate their ability to reach the phagosomes that harbor *M. tuberculosis*. This co-localization would allow the antibacterial agent to act on the pathogen immediately, because the acidic pH inside the phagosome would gradually dissociate the drug from its carrier [34,35] and create a high local concentration of the compound precisely at the site of bacterial residence. In the present study we found that NDs co-localized with *M. tuberculosis* in TB lesions and occupied the same cells and intracellular compartments—namely, phagosomes/phagolysosomes and lipid droplets. Using conventional and analytical transmission electron microscopy, we detected NDs within foamy macrophages of the lungs in *M. tuberculosis*-infected mice. These results indicate that hydrogenated NDs constitute a promising platform for the development of targeted anti-tuberculosis therapeutics.

Previously, other investigators demonstrated co-localization of biomedical nanoparticles with *M. tuberculosis* in a series of in vitro studies. In those investigations, composite nanoparticles based on iron oxide, graphene oxide, or selenium—each conjugated with rifampicin or isoniazid—accumulated within macrophages together with *M. tuberculosis* and exhibited antibacterial activity that exceeded that of free rifampicin or isoniazid [36,37,38,39].

Another promising drug delivery carrier is biodegradable metal–organic framework structures (MOFs). We have shown that these nanoparticles are efficiently internalized by Chlamydia-infected murine macrophages RAW264.7, co-localizing with the pathogens in the same phagosomes and exerting a measurable antibacterial effect [40].

Multi-walled carbon nanotubes conjugated with isoniazid and fluoxetine have shown potent antibacterial activity against *M. tuberculosis* in A549 and THP-1 cells in vitro [41]. Silver nanoparticles likewise display antibacterial properties and are being explored as a platform for formulating composite antibacterial agents [42]. However, for these nanoparticle types, intracellular co-localization with mycobacteria has not yet been demonstrated.

Previously, we demonstrated that in the lungs of uninfected mice, at early time points after administration (7 days), NDs are internalized by endothelial cells and interstitial macrophages [15]. In the present study, we showed that 30 days after administration, NDs are detected in interstitial macrophages located within alveolar walls (in regions of the respiratory portion of the lung that retain intact architecture) as well as in foamy macrophages within areas of TB-associated inflammation. Within inflammatory infiltrates, NDs are found in the same cells as *M. tuberculosis*—namely, foamy macrophages—which represents an important finding of this study.

The exploratory cell-level analysis suggested that ND distribution in infected lungs was associated with the cellular composition of inflammatory lesions. In inflammatory regions, NDs were frequently detected in foamy macrophages, including most foamy macrophages containing visible *M. tuberculosis*. This observation suggests that NDs can reach bacillus-containing macrophages within the inflammatory compartment. However, ND-positive foamy macrophages were also observed in the absence of detectable bacilli, and the presence of visible *M. tuberculosis* was not significantly associated with ND detection at the cell level. This may be due to the limitations of the transmission electron microscopy method, which uses ultrathin sections (60–80 nm) without full 3D reconstruction of the cell ultrastructure. Therefore, these findings should not be interpreted as clear evidence of selective targeting of bacillus-containing macrophages. Rather, ND accumulation may reflect uptake and retention by phagocytic foamy macrophages, which are abundant in inflammatory lesions.

In adjacent aerated alveolar regions, ND-positive cells were detected less frequently, whereas *M. tuberculosis*-positive cells were not identified. These observations indicate that ND localization in infected lung tissue may be influenced by the local inflammatory microenvironment and the distribution of phagocytic cell populations. Thus, NDs appear to access macrophage-rich inflammatory niches, including bacillus-containing foamy macrophages, while their accumulation is likely related primarily to macrophage phenotype rather than to the visible presence of bacilli. Foamy macrophages are known to have a large number of different phagocytic receptors on the membrane surface [43], and many of them are associated with the absorption of nanoparticles of various origins [44].

NDs accumulate predominantly within phagosomes/phagolysosomes and only occasionally appear as inclusions within lipid droplets; thus, their intracellular localization largely coincides with that of *M. tuberculosis*. The size and chemical properties of hydrogenated NDs allow them to accumulate within cells via endocytosis (phagocytosis) and subsequently traffic to phagosomes containing *M. tuberculosis*. Importantly, the inhibition of phagosome–lysosome fusion characteristic of *M. tuberculosis* infection [45], does not appear to prevent NDs from entering phagosomes that harbor the bacteria, as demonstrated by micrographs showing their co-localization within the same intracellular compartment. Thus, our findings demonstrate not only a certain tropism of hydrogenated NDs toward lung tissue but also their pronounced tropism toward macrophages containing intracellular pathogens.

Foamy macrophages contain numerous electron-dense structures because these cells actively generate phagosomes/phagolysosomes and lipid droplets, organelles that are heterogeneous in composition and may stain unevenly with heavy-metal reagents. In addition, artefacts related to contrasting can arise during sample preparation [46] and may be mistaken for nanoparticles. For this reason, in every case in which intracellular co-localization of NDs and *M. tuberculosis* was observed on ultrathin sections, the identity of the nanoparticles was unequivocally confirmed by electron diffraction analysis, thereby eliminating the risk of a false-positive localization of NDs. The obtained results on ND co-localization with *M. tuberculosis* raise the question of the advisability of further conjugating these nanoparticles with anti-TB drugs.

Recent studies have shown that nanodiamond surfaces can be chemically modified, allowing the subsequent conjugation of therapeutic agents [47]. Anti-tuberculosis drugs span both hydrophobic and hydrophilic classes: rifampicin [48], bedaquiline [49] and pretomanid [50] are hydrophobic, whereas isoniazid [51] is hydrophilic, and linezolid is considered moderately lipophilic as well as moderately hydrophilic [52]. Unlike carboxylated NDs, hydrogenated NDs exhibit predominantly hydrophobic surface properties [53], a feature that may account for their entry into lipid droplets together with *M. tuberculosis*. This finding provides an important rationale for developing ND-based anti-TB formulations for targeted delivery to infected lung macrophages, since most anti-TB agents are likewise hydrophobic. We anticipate that conjugates of these drugs with the hydrogenated NDs employed in the present study will display comparable behavior and will co-localize with *M. tuberculosis* in foamy macrophages—an aspect that will be addressed in future work.

In 2014, it was shown that oxidized HA can exhibit antibacterial properties even without conjugation with a drug [54]. There is also a point of view according to which the ability of NA to adhere to bacterial cells can temporarily reduce the rate of their growth, which is mistakenly taken for the antibacterial effect of the particles themselves [55]. In our work, we did not observe the death of mycobacteria when they were colocalized with nanodiamonds, which may presumably be due to a different functionalization of the particle surface. It should also be noted that there are currently no studies demonstrating the biodegradation of diamond nanoparticles in cells and tissues. In our study, we also observed no signs of their degradation or significant transformation in cellular phagosomes. It is possible that some of the nanodiamonds that enter the body will be eliminated from the respiratory tract along with mucus after the natural death of the carrier cells, but most likely a significant amount of them will remain in the lungs and other organs (liver and spleen). However, liver, spleen and lung cells containing ND did not show any pathological signs at the ultrastructural level (with the exception of pulmonary foamy macrophages, which in themselves are a sign of infection; their formation is not associated with ND) [43]. This allows us to continue to consider NA as a platform for efficient drug delivery to foamy macrophages, despite the possibility of their long-term presence in the lungs after dissociation of the proposed drug.

## 5. Conclusions

This study is relevant to the development and implementation of nanotechnology achievements for medical use, one of the most important areas of which is overcoming the resistance of bacteria and their complex communities (biofilms), including MDR/XDR M. tuberculosis.

In our study, we evaluated the colocalization of hydrogenated nanodiamonds, as a potential drug delivery platform, with *Mycobacterium tuberculosis* strain H37Rv. Our results demonstrate that, in the area of TB-specific inflammation in the lungs of infected mice, nanodiamonds are detected only within foamy macrophages. An important addition to this result is the fact that *M. tuberculosis* also localizes specifically in these cells. We did not detect the presence of NA in other cells within the inflammation area, unlike in the alveoli. This fact indicates selective accumulation of these nanoparticles, likely due to the properties of the foamy macrophages themselves and the microenvironmental conditions that develop during TB inflammation. However, no signs of cytotoxic effect of nanodiamonds in liver, spleen, and intact lung cells were detected in vivo. We also found no signs of toxic effects of ND on *M. tuberculosis* in foamy macrophages when they were colocalized in the same phagosomes. These data may also indicate the safety of ND and their aggregates for living systems. However, questions regarding the nanobiosafety of ND themselves for cells and tissues, as well as the possibility of their biodegradation and elimination from the body after longer exposure periods, remain open. Further research in this area, including the use of ND conjugates with anti-TB drugs, will provide answers to these questions. These findings position nanodiamonds as a highly promising carrier for the targeted delivery of anti-TB therapeutics to the lungs during tuberculous inflammation.

## Figures and Tables

**Table 1:** Exploratory cell level quantitative analysis of ND and *M. tuberculosis* distribution in inflammatory and aerated alveolar regions of infected mouse lungs.

Parameter in Inflammatory Regions	Count	Percentage	95% CI
Foamy macrophages among all counted cells	424/3586	11.8%	10.8–12.9%
ND + foamy macrophages among all counted cells	350/3586	9.8%	8.8–10.8%
ND + foamy macrophages among foamy macrophages	350/424	82.5%	78.6–85.9%
*M. tuberculosis* + foamy macrophages among foamy macrophages	273/424	64.4%	59.7–68.8%
Foamy macrophages containing both NDs and *M. tuberculosis* among foamy macrophages	220/424	51.9%	47.1–56.6%
ND + cells among *M. tuberculosis* + foamy macrophages	220/273	80.6%	75.5–84.8%
Parameter in aerated alveolar regions	Count	Percentage	95% CI
ND + cells among all counted cells	87/1240	7.0%	5.7–8.6%
*M. tuberculosis* + cells among all counted cells	0/1240	0%	0–0.31%

Proportions are presented with 95% confidence intervals calculated using the Wilson score method.

## Data Availability

The original contributions presented in this study are included in the article/Supplementary Material. Further inquiries can be directed to the corresponding author.

## Supplementary Materials

The following supporting information can be downloaded at https://www.mdpi.com/article/10.3390/pharmaceutics18060671/s1: Figure S1. ND aggregates in macrophages and hepatocytes of the liver of M. tuberculosis-infected mice; Figure S2. ND aggregates in splenic macrophages of M. tuberculosis-infected mice.
