# Supplementary material for: Nanodiamonds Co-Localize with Mycobacterium tuberculosis in Foamy Macrophages of Infected Mouse Lungs
Source: Pharmaceutics. 2026 May 29;18(6):671. doi: 10.3390/pharmaceutics18060671 (PMC13306093; doi:10.3390/pharmaceutics18060671)
Supplement: Supplementary file 1 [file pharmaceutics-18-00671-s001.zip › pharmaceutics-4257682-supplementary.pdf]

Supplementary figures

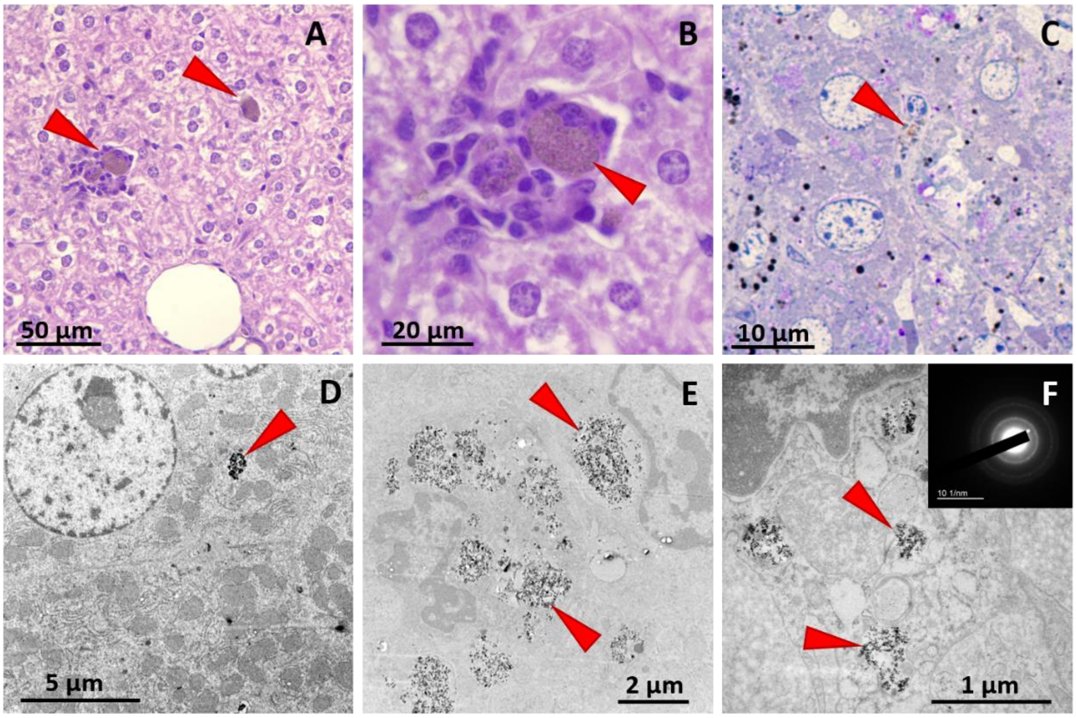

Figure S1. ND aggregates in macrophages and hepatocytes of the liver of *M. tuberculosis*-infected mice. A – General view of liver cells showing dark ND aggregates; hematoxylin–eosin staining, light microscopy. B – Higher-magnification view of liver cells showing dark ND aggregates; hematoxylin–eosin staining, light microscopy. C – Kupffer cells containing ND aggregates; semithin sections, azure B and basic fuchsin staining, light microscopy. D–F – Liver cells containing ND aggregates, conventional TEM. D – Hepatocyte containing ND aggregates. E–F – Kupffer cells with large ND aggregates in phagosomes. The inset shows an electron micrograph of an ND aggregate obtained by analytical TEM. Red arrows indicate ND aggregates in phagosomes.

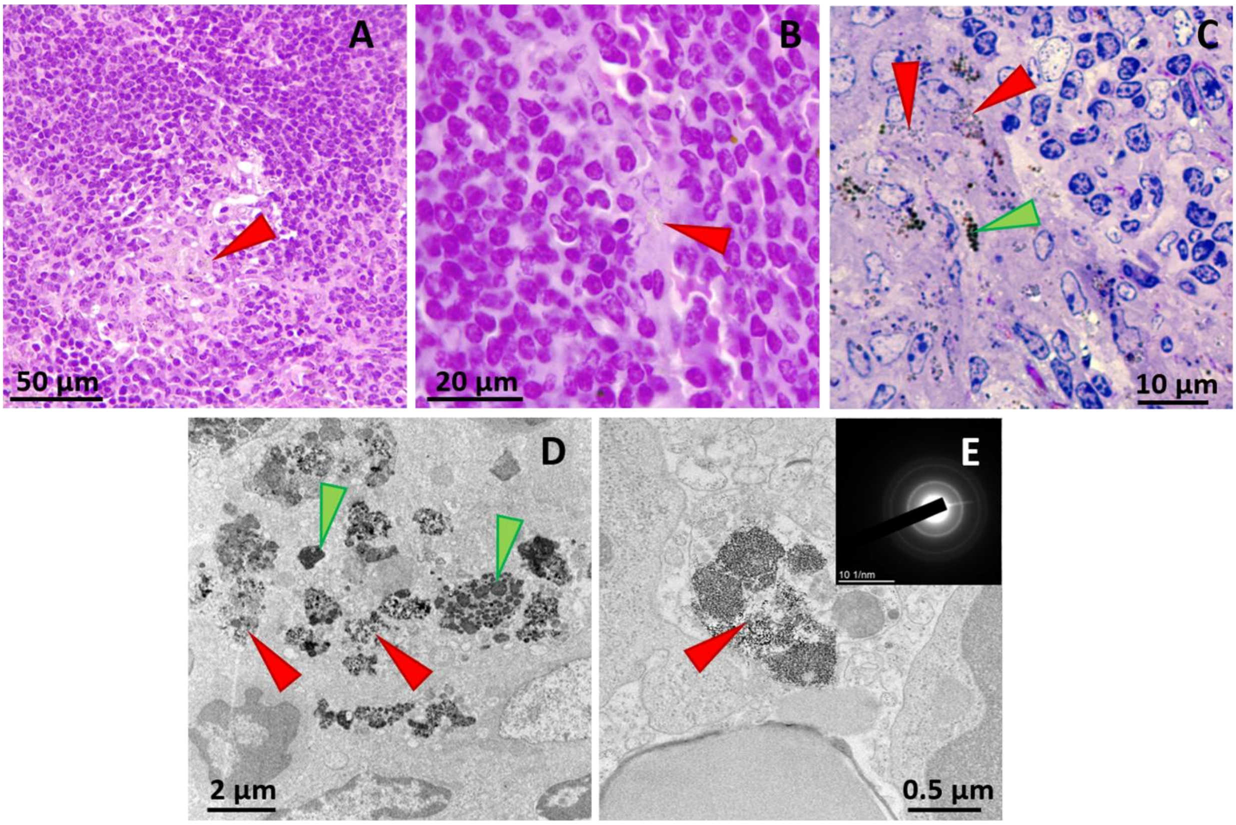

Figure S2. ND aggregates in splenic macrophages of *M. tuberculosis*-infected mice. A – General view of the marginal zone of a lymphoid follicle; hematoxylin–eosin staining, light microscopy. B – Higher-magnification view of the marginal zone of a lymphoid follicle showing a macrophage with ND aggregates; hematoxylin–eosin staining, light microscopy. C – Semithin sections showing foamy macrophages and macrophages containing ND aggregates; azure B and basic fuchsin staining, light microscopy. D–E – Splenic macrophages containing ND aggregates, conventional TEM. The inset shows an electron micrograph of an ND aggregate obtained by analytical TEM. Red arrows indicate ND aggregates in phagosomes; green arrows indicate hemosiderin aggregates and lipid droplets.
